# Effects of Diet and Exercise on Circadian Rhythm: Role of Gut Microbiota in Immune and Metabolic Systems

**DOI:** 10.3390/nu15122743

**Published:** 2023-06-14

**Authors:** Yidan Cai, Yanan Liu, Zufang Wu, Jing Wang, Xin Zhang

**Affiliations:** 1 Department of Food Science and Engineering, Ningbo University, Ningbo 315211, China; 2 China Rural Technology Development Center, Beijing 100045, China

**Keywords:** intestinal microbiota, circadian rhythm, exercise, immune system, metabolite

## Abstract

A close relationship exists between the intestinal microbiota and the circadian rhythm, which is mainly regulated by the central-biological-clock system and the peripheral-biological-clock system. At the same time, the intestinal flora also reflects a certain rhythmic oscillation. A poor diet and sedentary lifestyle will lead to immune and metabolic diseases. A large number of studies have shown that the human body can be influenced in its immune regulation, energy metabolism and expression of biological-clock genes through diet, including fasting, and exercise, with intestinal flora as the vector, thereby reducing the incidence rates of diseases. This article mainly discusses the effects of diet and exercise on the intestinal flora and the immune and metabolic systems from the perspective of the circadian rhythm, which provides a more effective way to prevent immune and metabolic diseases by modulating intestinal microbiota.

## 1. Introduction

The gut microbiota are mutually restricted and interdependent according to a certain proportion, showing a dynamic ecological balance as a whole. Intestinal flora can be divided into symbiotic bacteria, conditional pathogenic bacteria and pathogenic bacteria. As the main body of intestinal flora, they have a relatively fixed and stable state and reside in the intestine to jointly maintain the homeostasis of the body [1,2]. Research shows that the composition of intestinal flora changes with dietary intake, and the types of bacteria vary among people of different ages [3,4]. The composition of intestinal flora is also affected by host genetics [5], antibiotic use [6], lifestyle [7] and concomitant diseases [8,9].

Circadian rhythms refer to the continuous changes in the life activities of organisms with a cycle of about 24 h. Among them, the circadian oscillator is the central mechanism driving the circadian rhythm, which can keep the biological rhythm relatively consistent with the circadian rhythm. The generation of circadian rhythm is mainly regulated by the hypothalamic suprachiasmatic nucleus (SCN) and the biological-clock gene in the peripheral tissues [10]. In mammals, the circadian-rhythm axis is composed of the retina, SCN and pineal gland. Their interactions can produce and regulate physiological and behavioral rhythms in the organism. Among them, the SCN, also known as the circadian-rhythm pacemaker, plays a dominant role in the circadian rhythm, which can control the physiological and behavioral rhythms of the organism. At the same time, the peripheral-biological-clock system (mainly including muscle, adipose tissue, liver and intestine) also participates in regulating the circadian rhythm [11]. The biological-clock gene refers to the gene that can control the biological rhythm of organisms. It does not only regulate the sleep cycle and cognitive function of mammals, but also regulates most of their circadian-rhythm cycles in physiological conditions [12].

**Figure 1:**
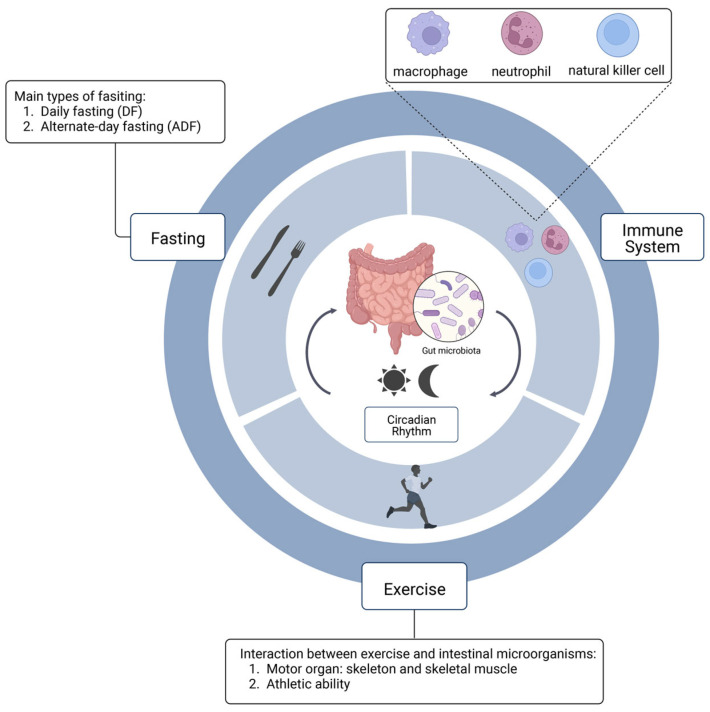
The interaction between gut microbiota and circadian rhythm.

A large number of studies have shown that the patterns of exercise and diet have a certain degree of impact on intestinal flora, as well as on the immune system and metabolic system of the human body. Depending on the different metabolic states of the host (normal individuals [13], diabetes [14], obesity [15], hypertension [16], anxiety and cognitive impairment [17]), exercise can have different positive effects on the intestinal flora to promote host health. Studies have shown that the circadian-rhythm oscillation between the activity of the immune system and the function of the immune system is consistent, and that an imbalance in the biological clock will lead to the abnormal response of the immune system. The circadian rhythm plays an important role in the physiological metabolism of the body, and the activity of the intestinal flora also participates in the metabolic process of its host [18,19]. In addition to effective weight loss, fasting can also reduce blood pressure, blood lipid level, improve brain health, enhance immunity, improve intestinal health and enhance circadian rhythm [20]. Fasting can reduce fat deposition, improve glucose tolerance and insulin sensitivity, as well as significantly improve the hyperglycemia and pancreatic island function of early diabetes patients, and can also have preventive and therapeutic effects on type 2 diabetes. In addition, fasting can also extend the life of mice [21], improve insulin sensitivity, and reduce fasting blood glucose and total cholesterol without losing weight [22]. This paper discusses the effects of diet and exercise on intestinal flora and the immune and metabolic systems from the perspective of circadian rhythm, so as to provide an effective way for the prevention of immune and metabolic diseases (Figure 1).

## 2. The Relationship between Circadian Rhythm and Gut Microbiota

The circadian rhythm of intestinal flora is closely related to the biological clock, which is an important mechanism to link individual physiological rhythms with external light and other factors [23]. The biological clock also has a universal influence on the regulation of body homeostasis. Its main function is to synchronize energy metabolism and behavior rhythm [24]. In the natural state, the biological clock receives the periodic signals of the external environment, regulates its own oscillating system through a feedback loop composed of biological-clock genes and their coded proteins, and keeps various life activities of the body synchronized with the environmental cycle [25]. The central-biological-clock system of mammals is located in the SCN, which is the “pacemaker” of the circadian rhythm. As the main pacemaker of the circadian rhythm, the SCN controls the rhythmic activities of the whole body. The oscillation of the clock autonomy is mainly generated by the “Transcription Translation Feedback Loop (TTFL)”, which is composed of a series of clock genes with a CLOCK-BMAL1 heterodimer as the core in the central-biological-clock system and the peripheral-biological-clock system [26,27].

The central-biological-clock system’s main function is to coordinate the physiological activities of the peripheral tissues. It keeps the peripheral-biological-clock system stable through a variety of nerve and humoral signals, thus enabling the body to adapt to the needs of the external environment. As a consequence, the intestinal flora belonging to the peripheral-biological-clock system will change due to changes in the central biological clock of the host. On the contrary, the imbalance of intestinal flora will affect the central biological clock to some extent, as well as the immune and nutritional-metabolism functions of the intestinal physiological state.

The disturbance of the host circadian rhythm will lead to the same consequences for intestinal flora, which will directly affect the intestinal immune and nutritional-metabolism functions. It was found that the expression profiles of nuclear receptors and biological-clock genes in the cecum and colon epithelial cells of GF mice were different from those of normal epithelial cells. The homeostasis of intestinal-microflora metabolites and the rhythmic expression of Per2 and Bmal1 genes in the hepatocytes of mice fed with a high-fat diet were affected [28]. Through the knockout test of mouse biological-clock genes Per1 and Per2, it was found that the disturbance of the host biological clock would indeed cause changes in the flora, thus indicating that changes in the circadian rhythm of intestinal flora would indeed be regulated by biological-clock genes [29]. By comparing the mRNA expression levels of Occludin and Claudin-1 in the bodies of wild-type mice and mice with the loss-of-function mutation of a key clock gene Per2, as well as the differences in colonic permeability, it was found that the intestinal epithelial barrier in mice with central-biological-clock disorder was destroyed, and the intestinal permeability was increased, which indirectly indicated that the biological-clock disorder could cause the disorder of intestinal flora [30]. Intestinal flora also plays a role in regulating the circadian rhythm of the host. Studies have proved that Histone Deacetylase 3 (HDAC3) is the key substance in intestinal flora that plays a role in regulating the circadian rhythm of the small intestine [31].

The peripheral-biological-clock system of mammals is mainly located in regions of the brain other than the SCN and in most cells and tissues of the whole body. The peripheral biological clock has its own biological rhythm, but it is also regulated by the central biological clock. Therefore, its physiological function is regulated by the clock gene’s expression and regulation of downstream clock-control genes. Studies have shown that there is a certain relationship between the intestinal flora and the peripheral biological clocks of different organs. The intestinal flora is closely related to the circadian rhythm of intestinal mucosal immunity in structure and function. When mice were fed with mixed antibiotics, the gene expression profile of the colonic epithelial cells changed significantly, indicating that the imbalance in the intestinal flora really caused changes in intestinal-related functions [32]. By detecting the expression profiles of nuclear-receptor and clock genes in the cecum and colon epithelial cells of mice treated with antibiotics, we found that their expressions were different from those in normal intestinal epithelial cells, which shows that intestinal flora participated in regulating the expression of nuclear-receptor and clock genes in intestinal epithelial cells [33]. The disorder of the intestinal biological clock may lead to the destruction of intestinal-mucosal-barrier function and inflammation, and cause imbalance in the intestinal flora.

Intestinal flora is also related to the biological-clock system of the liver. Both the intestine and the liver originate from the primitive intestine in the embryonic stage, and later develop into two complex and mature metabolic organs [34]. By analyzing the gene expression of liver samples, the mRNA expression pattern of the biological-clock gene in the liver of infertile mice was significantly different from that of SPF mice [35]. This shows that the loss of intestinal microbiota will change the expression of the liver biological-clock gene, which suggests that the change in the intestinal microbiota may cause the liver-biological-clock and metabolic-function disorder.

## 3. The Relationship between Exercise and Gut Microbiota

Intestinal microbiota is considered as the cornerstone to maintain the health of the human host. It not only determines the effectiveness of obtaining nutrition and energy from food, but also can produce a large number of metabolites to regulate the metabolism of the host. The intestinal microbiota can improve the bioavailability of amino acids and optimize the decomposition, absorption and metabolism of protein. In a human study involving excellent football players, it was found that the intestinal microbiota diversity index was positively correlated with protein intake [36]. Studies show that exercise is closely related to the circadian rhythm, and the benefits of exercise methods under different conditions are also different. The circadian rhythm of mice is changed when the mice are tested with wheel exercise under no light conditions. In addition, the time of exercise also involves the regulation of the physiological clock. Regular exercise will affect the circadian rhythm of skeletal muscle and lung, but it has little effect on the SCN [37]. Exercise not only enhances the expression of core-clock gene Per2, but also promotes the rhythmic movement of muscle cell Per2 phases [38]. In addition, research shows that exercise is not only an important factor in regulating the human body’s peripheral rhythm, but also that exercise ability is affected by the time of day. Relevant studies have found that, even if the environmental conditions and time arrangements are biased relative to the athletes’ training, the neuromuscular function, maximum oxygen uptake and grip strength are highest in the afternoon [39]. In addition, the exercise cycle seems to have a special impact on exercise ability. Compared with the random timing of exercise, fixed-time maximum equal-length autonomous-joint-contraction training can better improve the anaerobic exercise ability of skeletal muscle, that is, the maximum explosive force of muscle [40].

### 3.1. Possible Triggers of Cognitive Decline in the Process of Brain Aging

The skeleton is the basis of the human body’s shape, and also the main attachment points for skeletal muscle and an important part of the sports system. A large number of studies have proved that the intestinal flora has an important impact on bone metabolism using GF mice, antibiotic intervention, probiotic supplementation and other experiments, which have suggested that intestinal flora may be the main regulator of bone mass [41]. Relevant research results indicate that the stimulation effect of intestinal flora on bone anabolism may be mediated by insulin-like growth factor 1 (IGF-1) [42].

As the largest organ of the human body, skeletal muscle accounts for about 40% of body weight and is the direct functional tissue for completing exercise. Many studies have shown that the intestinal flora is related to skeletal-muscle metabolism and muscle-fiber type, and is mainly responsible for body movement, metabolism and the secretion of muscle factors to regulate other organs [43]. Short-chain fatty acids (SCFAs) are one of the important metabolites of intestinal flora. As signal molecules, they can affect a series of activities of the host, and they are also a key factor in regulating the physiological function of skeletal muscle [44]. When comparing the skeletal muscles of GF mice and SPF mice, it was found that GF mice had skeletal-muscle atrophy, and the transplantation of intestinal flora could significantly improve the degree of muscle atrophy in GF mice and improve the quality of their skeletal muscle. Supplementing SCFAs can partially alleviate skeletal-muscle injury, which proves that intestinal flora plays an important role in improving the quality of skeletal muscle in mice, and has the potential to treat skeletal-muscle injury [45].

It was found that long-distance endurance running caused significant changes in the abundance of some microbiota and 40 kinds of fecal metabolites in the intestinal environment, among which the difference between the metabolites of organic acids and of nucleic acids was the largest [46]. Intestinal microbiota provides essential metabolites for skeletal-muscle mitochondria, and can regulate key transcription coactivators, transcription factors and enzymes involved in mitochondrial biogenesis [47]. Among them, SCFAs, the final products of the microbial degradation of intestinal carbohydrates, constitute the link between food, intestinal microorganisms and host material energy metabolism. Exercise can also improve the circadian-rhythm disorder of skeletal muscle, in which the remodeling of skeletal muscle is a key component of the organism’s response to environmental changes. Exercise can lead to changes in muscle structure, circadian rhythm, and physiological and behavioral fluctuations. The duration is about 24 h, which is maintained by the core-clock mechanism. Exercise-induced remodeling and circadian rhythm depend on the transcriptional regulation of key genes [48]. Exercise intervention will affect the molecular-clock mechanism of human skeletal muscle, including a significant increase in Bmal1 gene expression and Per2 protein expression in skeletal muscle [49]. In human studies, the rhythmic gene expression of Rev-erbα/β, Sirt1 and Nampt was found in primary cells extracted from endurance-training athletes, while it was not found in the primary cells of sedentary people [50], which indicated that exercise can improve the disorder of the circadian rhythm of skeletal muscle to a certain extent. Studies have shown that early-morning training promotes the advance of the circadian-rhythm phase, while night training induces the delay of the circadian-rhythm phase [51,52]. In the study of nocturnal rodents, it was also found that wheel or treadmill exercise would change the expression of the core-clock gene of skeletal muscle, which might affect the circadian rhythm [53]. In a long-term experiment of autonomously rotating mice, it was found that the peak transcriptional expression of genes related to circadian-rhythm regulation in mice showed an increasing trend [54]. In addition, the elderly can relieve the circadian-rhythm disorder caused by aging by persisting in aerobic exercise for more than half an hour every day [55]. It can be seen from the above studies that exercise can regulate the core-clock mechanism in skeletal muscle, and exercise time can change the phase of the circadian rhythm, thus relieving disorder of the circadian rhythm of skeletal muscle.

### 3.2. Intestinal Microbiota and Exercise Ability

**Figure 2:**
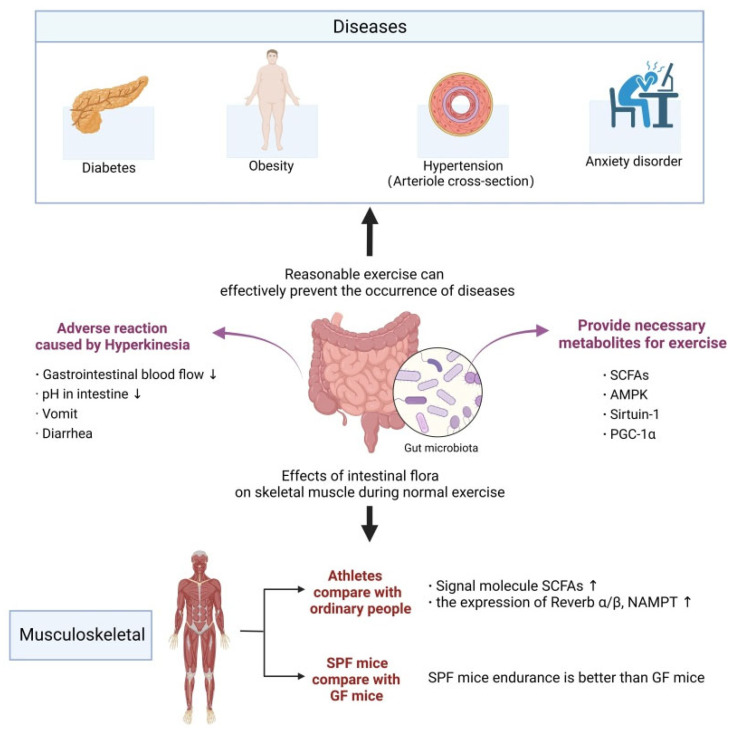
The effects of reasonable exercise for disease prevention through intestinal microbiota. “↑”: Increase, “↓”: Decrease.

Intestinal flora is not only one of the mediating factors of sports health effects, but it also participates in the occurrence of sports stress response and sports fatigue, affecting the body’s sports ability [56,57]. It was observed that for sterile mice of a Parkinson’s model, their motor dysfunction symptoms were lighter than those with the normal flora, but the motor-dysfunction symptoms were significantly aggravated after the transplantation of the flora of Parkinson’s disease patients, indicating that the intestinal flora participated in regulating the motor dysfunction caused by Parkinson’s disease [58]. Hsu et al. proved for the first time that there was a potential relationship between intestinal flora and exercise ability, and their study found that SPF mice and mice colonized with fragile Bacteroides had longer endurance-swimming times than GF mice [59]. Later, Denou et al. obtained similar results [60]. Consequently, it can be concluded that intestinal flora has a potential application value in delaying exercise fatigue and improving exercise ability (Figure 2).

During strenuous exercise, the body redistributes blood from the visceral circulation to respiratory and muscle tissues, and prolonged insufficient blood supply can easily cause vomiting, abdominal pain and diarrhea [61]. Exhaustive exercises can significantly affect the level of lipid metabolism and lipid peroxidation, and abnormal lipid metabolism is the main factor inducing coronary heart disease. It has been reported that exhaustive exercise can change the diversity and micro-ecological structure of intestinal flora. Stable bacterial population structure may be one of the important conditions for maintaining health and stable sports performance during exhaustive exercise [62].

## 4. Immune System and Gut Microbiota

At present, microbial preparations such as bacterial products, bacterial toxins, probiotics, etc., have been used in the reconstruction of immune tolerance in clinical practice [63]. Intestinal flora has been proved to promote tolerance to autoantigens, regulate intestinal movement and secretion, maintain the integrity of intestinal mucosal barrier and maintain the normal activities of the immune system [64]. The existence of intestinal mucosa can separate the internal and external environment of the human body, form an “intestinal barrier structure”, prevent the passage of antigens, toxins and microorganisms, and ensure the immune tolerance of the body to food antigens and intestinal flora [65]. SCFAs have functions of anti-inflammation, regulating the disorder of glucose and lipid metabolism, and improving therapeutic effects on tumors [66]. SCFAs are generally produced by the fermentation of dietary fiber, including acetate, propionate and butyrate, among which butyrate is the main energy source of colon cells and is related to reducing the risk of colorectal cancer [67].

The circadian-rhythm disorder caused by shift work can affect the expression of serum cytokines in SD rats, which provides a theoretical basis for circadian-rhythm disorder affecting the immune system function of the body. Macrophages, neutrophils and NK cells are relatively more studied in the innate immune system.

Macrophages, as immune cells, also have a molecular biological clock. The phagocytosis of macrophages and their expressions of cytokines and chemokines all show obvious circadian rhythm. One study found that the phagocytosis of macrophages changed with the time of a day. Under the light/dark cycle (LD), the phagocytosis of peritoneal macrophages obtained from mice in the late light period was stronger than that in other time periods in the course of a day [68]. Another study found that the level of cytokines expressed by macrophages after being stimulated by endotoxins showed a significant circadian rhythm. Tumor necrosis factor being secreted by macrophages cultured in vitro at different time points after being stimulated with tumor necrosis factor α (TNF-α) and IL-6 has an obvious circadian rhythm [69]. Studies on the biological-clock gene of macrophages have shown that the expression patterns of the biological-clock gene Bmal1 in their cells is similar to that in SCN and peripheral-biological-clock tissues.

Neutrophils mainly migrate to injured tissues in the early stage of inflammation, and they participate in phagocytosis and secrete anti-inflammatory cytokines. Neutrophils do not only play an immune role in bacterial and fungal infections, but also in many autoimmune diseases. More and more studies have shown that the function change of neutrophils is related to time. Under the LD cycle, the level of neutrophils in the peripheral cycle shows a low-amplitude oscillation throughout the day, and it reaches the peak at the beginning of the quiescent period [70]. It is worth noting that the reduced activity of neutrophils will lead to serious infectious diseases, and inappropriate activation of neutrophils will also produce various autoimmune and inflammatory diseases. It has also been found that the migration of neutrophils can be indirectly regulated by controlling the expression of endothelial chemokines and adhesion molecules through the activation of β-adrenaline receptors [71].

NK cells are the key component of innate immunity, which can resist fungal, bacterial and viral infections. Studies have shown that NK cytotoxicity will show the characteristics of circadian rhythm, and the cytotoxicity is the greatest in the dark period. The research on the mechanism and physiology of NK-cell rhythm has increased significantly in recent years. Splenic NK cells have a functional molecular-clock mechanism and its core-clock gene follows the 24 h oscillation law of the circadian rhythm [72]. The cytolytic activity of NK cells has a circadian rhythm, and the expression of cytolytic factors and cytokines also presents a 24 h oscillation law.

Under the LD cycle and steady-state conditions, the expression level of the core-clock gene in B cells was detected, and it was found that there was a molecular-clock mechanism in B cells. Similarly, CD4^+^T cells purified from human blood at different time points in the first day showed the existence of the core-clock gene [73]. In human blood samples, the number of T cells generally increased at night, decreased in the morning, and maintained a low level during the day [74]. The concentration of cortisol and catecholamine in plasma showed obvious diurnal oscillation, which showed that the diurnal concentration was the highest and the nocturnal concentration was the lowest. It has been found that peripheral-blood cortisol and catecholamine regulated the rhythmic change in the number of T-cell subsets through the concentration difference in different time periods [75]. The number of primitive T cells and CD4^+^T and CD8^+^T cell subsets had the lowest diurnal rhythm, while the number of effector CD8^+^T cell subsets reached the peak during the day [73].

## 5. Metabolism and Gut Microbiota

The biological clock can change intestinal permeability. A mutant host biological-clock gene caused an increase in intestinal mucosal permeability, which led to an imbalance in the intestinal flora, and finally aggravated the lipid metabolism disorder and increased the incidence of fatty liver [76]. The mice were given a high-fat and high-sugar diet and the day and night were reversed. Gene detection of their colon biological clock and 16R rRNA sequencing of their feces showed that circadian rhythm changes in Per2 expression and composition and structure changes in their intestinal flora were found, respectively [77]. The clock gene Bmal1 has anti-inflammatory effects, so the absence of the clock gene Bmal1 will lead to changes in the flora phenotype [78]. The change in feeding rhythm will interfere with the flora, which will reduce the content of Bacteroides in the flora and increase the content of Firmicutes, which will increase the incidence of obesity [76]. The clock gene Bmal1 can play an anti-inflammatory role in mice. When Bmal1 is absent, the composition and structure of the flora will change to an inflammatory phenotype. The increase in bacteria such as Rikenellaceae and Clostridiaceae is closely related to the occurrence and development of ulcerative colitis and Crohn’s disease [78]. All the above results indicate that the change of the host biological clock will lead to imbalance in the flora, thus causing a variety of metabolic diseases. In addition, the disorder of the circadian rhythm of the intestinal flora will change the transcript collection of the host biological-clock gene, destroy the stability of amino acids, polyamines and other metabolites in the serum, and affect the detoxification function of the liver, which directly indicates that the disorder of the intestinal flora leads to the change in the expression of the liver biological-clock gene, and then leads to the disorder of the liver biological clock and the destruction of the metabolic function. In conclusion, the interaction between flora rhythm and host rhythm plays an important role in the immune system.

Studies have found that intestinal flora can participate in lipid metabolism by regulating the expression of the host Nfil3 gene under physiological conditions. The Nfil3 gene is an important link between flora and circadian rhythm and host metabolism [79]. Metabolites of Gram-negative bacteria (flagellated) can activate dendritic cells (DCs) through Toll-like receptor (TLR) and downstream MyD88 pathways. DCs secrete interleukin-23 (IL-23) to make congenital lymphocyte 3 (ILC3) secrete IL-22, and finally act on intestinal epithelial cells to activate the STAT3 pathway in cells. Activated STAT3 inhibits clock-gene transcription receptor Rev-erbα. The expression of Nfil3 is up-regulated, which promotes the metabolism and storage of lipid substances. On the contrary, when the stability of intestinal flora is destroyed, the biological rhythm of the host will be changed, and the expression of Nfil3 will be down-regulated, and thus the lipid metabolism will be disturbed [80]. As a result, the intestinal flora signal is transmitted to the host epithelial cells through the DC-ILC3-STAT3 pathway, thus determining the key pathway that the intestinal flora and its host affect metabolism by regulating biological rhythm.

## 6. Intermittent Fasting and Intestinal Flora

Intermittent fasting (IF), also known as time-limited diet, is a popular dietary intervention method, mainly for the purpose of prolonging the fasting time, which does not affect the food structure gradually formed with the growth in individual life. At present, IF schemes have mainly two types which have been applied to rodents, namely, daily fasting (DF) and alternate-day fasting (ADF). For daily fasting, the duration of fasting varies from 12 to 20 h. There is a time window of 16 h for fasting and the following 8 h for eating (16:8), or the proportion of 20 h for fasting and the following 4 h for eating (20:4), or other times. In alternate-day fasting, including 24 h fasting (no calorie intake) and subsequent 24 h eating (free food and water consumption), the choice of fasting frequency is different. For example, the 5:2 strategy is to mix two-day fasting with five-day non-restrictive eating, as well as other eating frequencies [81].

Animal and human studies have shown that many of the health benefits of IF are not only a reduction in the production of free radicals or weight loss, but also that it triggers evolutionarily conservative and adaptive cellular responses that are integrated into the organs in a way that improves glucose regulation, increases compression resistance and inhibits inflammation [82,83]. During fasting, cells will enhance their defenses against oxidative and metabolic stress, and activate the pathways to remove or repair damaged molecules [84]. One study shows that when rats start an ADF program from a young age, their average life span can be extended by up to 80%. However, the degree of influence of caloric restriction on a healthy life span and total life span varies, and it may be affected by gender, diet, age and genetic factors [85].

Studies have shown that fasting and caloric control can change intestinal flora, increase insulin sensitivity, reduce the expression of inflammatory factors related to lipid metabolism, and thus prevent metabolic diseases [86,87]. On the contrary, intestinal flora can enhance the intake of food energy, improve the synthesis of fatty acids, and promote the deposition of fat droplets in the liver and adipose tissue to cause obesity symptoms [88]. In addition, a research method based on sequence analysis showed that there is a significant difference between the intestinal flora of fasting and non-fasting individuals [89]. For instance, a diet program of fasting every other day can significantly increase the OTU abundance of Firmicutes in the feces of male C57BL/6N mice, while reducing the abundance of most other phyla [22]. After a fasting intervention in female C57BL/6J mice, the fecal samples of these mice showed an increase in the abundance of Lactobacillus, Bacteroides and *Prevaliaceae* [90]. In addition, under an IF-treatment scheme for diabetes mice, the enrichment of Lactobacillus and reduction of *Akkermannia* was seen [91].

Relevant studies have shown that the overnight-fasting treatment can significantly reshape the intestinal microbial community of mice, and increase beneficial metabolites of microorganisms, thereby improving cognitive function. After 28 days of intermittent fasting in diabetes mice, it was found that a behavioral disorder could be improved through the metabolites of the microflora–brain axis: fasting enhanced the expression of mitochondrial-biogenesis and energy-metabolism genes in the hippocampus, reconstructed the intestinal microflora and improved the microbial metabolites related to cognitive function [92].

IF can improve obesity, insulin resistance, dyslipidemia, hypertension and inflammation [93]. In mouse models, studies have shown that IF can enhance cognition in many fields, including spatial memory, associative memory and working memory [94]. In addition, IF also can improve cognitive impairment caused by diabetes [92]. IF can improve many indexes of animals and humans, including blood pressure, high-density lipoprotein and low-density lipoprotein, cholesterol, TG, glucose, insulin level and insulin resistance [95]. IF can reduce the markers of systemic inflammation and oxidative stress related to atherosclerosis [96]. In addition, IF can also promote the production of tauroursodeoxycholate (TUDCA), the metabolites of intestinal microorganisms, by remodeling the intestinal flora of diabetes mice, thereby promoting the activation of TGR5, the receptor of TUDCA, and down-regulating the TNF-α mRNA expression of β-lactamase protects the retina of diabetes-model mice [91]. In a randomized trial to examine the effect of ADF on patients with non-alcoholic fatty liver disease (NAFLD), the results showed that ADF was a safe and tolerable diet for NAFLD patients, and it would reduce weight, fat content, total cholesterol and triglycerides [97]. Therefore, exploring the influence of different fasting methods on intestinal flora has a positive role in the prevention and treatment of obesity.

Intestinal flora ferments plant polysaccharides to produce SCFAs, which can also promote the maturation of the host immune system and fight against infection. Intestinal flora can directly or indirectly regulate the development of the intestinal mucosal immune-system components [98]. Intestinal flora and metabolites can interfere with intestinal epithelial cells to affect dendritic cells and macrophages through the epigenetic mechanism driven by the intestinal flora. Metabolites of the intestinal flora can induce the production of T regulatory cells and participate in mucosal tolerance. Intestinal flora can also induce B cells to mature, change their immunoglobulin subtypes, promote the activation of basophils and mast cells through immunoglobulin E instead of immunoglobulin A, and produce optimized intestinal flora. The intestinal flora is a key component of the digestive system, which can decompose complex carbohydrates and proteins, and to a lesser extent, can decompose the fat in the lower gastrointestinal tract. This process will produce a large number of microbial metabolites, which can play a role in the local or whole body after being absorbed into the blood stream. The rhythmic occupation of niches by certain species of microorganisms may be the cause of this metagenome fluctuation, and these niches can respond to the feeding or starvation stage. In addition, research also shows that mice with Per1 and Per2 gene knockout can promote the recovery of the circadian rhythm of the intestinal flora by restoring the feeding rhythm [99]. The results showed that the rhythmic fluctuations of some bacterial populations in obese mice caused by dietary problems could be restored by limited-time feeding. The functions of different microorganisms are related to the rhythm of eating. It is mainly because the eating rhythm can regulate the changes in the utilization of nutrients. The cell development, energy absorption and response functions after DNA damage of microorganisms mainly occur in the period when nutrients can be digested and absorbed, and the detoxification function mainly occurs in the non-eating period.

In mammals, the food signal is the most effective biological-clock timing signal other than the light signal in the environment. The regulation of the eating rhythm can induce the expression of the biological-clock gene in intestinal tissue, leaving it out of the control of the central SCN. That is to say, the host’s eating signal can participate in the formation of the circadian rhythm of the intestinal flora independently of the control of the central rhythm, and rhythmic eating can lead to the occurrence of a rhythmic oscillation of microbial flora [100]. The pattern and content of diet are considered to be the most important driving factors for shaping intestinal flora in a short time. In the long run, diet is also the most effective and healthy way to regulate intestinal flora.

## 7. Conclusions

Interventions in diet and exercise can effectively regulate imbalances in the circadian rhythm or the intestinal flora. Healthy and reasonable eating habits can help the intestinal tract to maintain a balance with the circadian rhythm, especially to control the rhythmic fluctuations of specific bacteria that cause obesity. The combination of regular physical exercise and a healthy diet can promote the generation of beneficial metabolites in the intestine, maintain the stability of the intestinal environment, repair the intestinal barrier, and then help prevent and treat diseases. However, the research on the mechanisms of the related flora and the effects of exercise and fasting is still relatively limited, and it is necessary to combine the two to conduct a systematic study on the body. Dietary survey methods can be used to intervene and monitor diet, and questionnaire surveys, activity-recorder measurements and mechanism analysis can be used to monitor exercise, in order to study the impact of daily diet and exercise in a more comprehensive manner. Therefore, we should formulate treatment plans corresponding to the patient population through interventions in diet and exercise, so as to provide a theoretical basis for later treatment of various immune, metabolic and gastrointestinal diseases.

## Data Availability

Not applicable.
